# Multiple Evanescent White Dot Syndrome After mRNA COVID-19 Vaccination

**DOI:** 10.7759/cureus.103227

**Published:** 2026-02-08

**Authors:** Risaki Sakamoto, Yoshio Hirano, Shuhei Tomita, Ryota Ando, Tomohiro Obayashi, Shuntaro Ogura, Tsutomu Yasukawa

**Affiliations:** 1 Department of Ophthalmology and Visual Science, Nagoya City University Graduate School of Medical Sciences, Nagoya, JPN

**Keywords:** coronavirus disease 2019 (covid-19), covid-19, covid-19 vaccination, multiple evanescent white dot syndrome, optical coherence tomography, steroid, vaccine

## Abstract

A 46-year-old woman noticed blurred vision in her right eye two weeks after receiving the fourth dose of a COVID-19 vaccine and visited a local eye clinic. Then, the patient was referred to our hospital to investigate the cause of decreased vision in the right eye. At the initial visit to our hospital, the best-corrected visual acuity (BCVA) in the right eye was 0.7 in decimal units and 1.5 in the left eye, respectively. Intraocular pressure was 17 mmHg in both eyes. Abnormal findings were observed only in the right eye; the left eye was completely normal. Therefore, all abnormal findings described below are related to the right eye. Anterior segment examination revealed inflammatory cells in the anterior chamber. The lenses in both eyes were almost clear, and the right eye showed vitreous cells and vitreous opacity. Fundus examination revealed multiple small white exudative spots. Optical coherence tomography (OCT) demonstrated disruption of the ellipsoid zone (EZ) and hyper-reflective lesions beneath the retina. Fluorescein angiography (FA) showed hyperfluorescence consistent with staining at the sites of exudative lesions, while indocyanine green angiography (ICGA) revealed corresponding hypofluorescent spots due to blocked fluorescence. Full-field electroretinography (ERG) showed reduced amplitudes of both a- and b-waves. Goldman perimetry identified a central scotoma in the right eye. Based on these findings, the patient was diagnosed with multiple evanescent white dot syndrome (MEWDS) in the right eye. Topical betamethasone sodium phosphate and fradiomycin sulfate were initiated six times a day for the right eye at the first visit. The day after the initial visit, the BCVA in the right eye rapidly declined to 0.01 in decimal units, prompting the initiation of oral prednisolone at 30 mg/day. Eight days later, a sub-Tenon’s capsule injection of triamcinolone acetonide (20 mg/0.5 ml) was administered. The symptoms gradually improved thereafter, and the hyper-reflective subretinal lesions observed on OCT disappeared. Oral steroid therapy was tapered, and the visual acuity (VA) improved to 1.0 in decimal units four months later. A case of MEWDS occurring after COVID-19 vaccination showed favorable recovery with oral steroid therapy and sub-Tenon’s injection.

## Introduction

COVID-19 was first reported in China in December 2019 and subsequently caused a global pandemic. In 2020, the World Health Organization declared COVID-19 a Public Health Emergency of International Concern. Vaccination with newly developed mRNA COVID-19 vaccines began worldwide in late 2020 and has been highly effective in controlling the pandemic. However, various adverse events following vaccination have been reported [1,2]. Among these, ophthalmic adverse effects, including inflammatory ocular diseases, have been described [3-5]. Although rare, cases of multiple evanescent white dot syndrome (MEWDS) occurring after COVID-19 vaccination have been reported [6-12]. MEWDS is an idiopathic disorder characterized by multifocal exudative lesions in the retina and choroid [11]. It is considered a part of the white dot syndromes, a group of entities presenting with multiple scattered white lesions in the posterior pole, and typically affects young adults, causing symptoms such as blurred vision and decreased visual acuity (VA) [13]. Although a causal relationship between MEWDS and COVID-19 vaccination remains unclear, we encountered a case of MEWDS that developed following COVID-19 vaccination [13]. MEWDS is generally known to have mild visual impairment and often undergoes spontaneous remission [14]. However, in the current case, significant visual loss was observed, but visual function improved after oral steroid therapy and a sub-Tenon’s capsule injection. We report this case because of its notable clinical course.

## Case presentation

**Figure 1:**
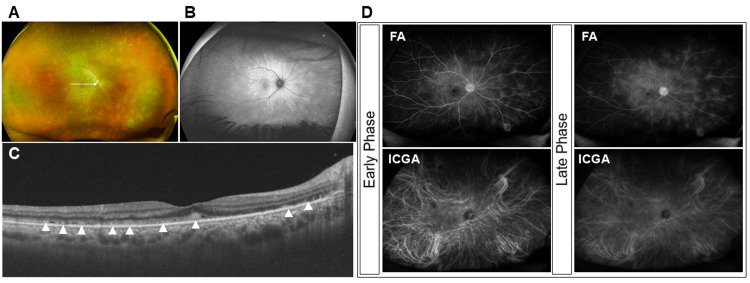
Baseline ocular findings A. Ultra-widefield (UWF) fundus photograph taken with Optos California (Nikon Corporation, Tokyo, Japan). Vitreous cells and vitreous opacity were observed. Multiple white exudative lesions in the right eye were observed. B. UWF fundus autofluorescence. The lesions corresponding to the white exudative spots exhibit a slightly reduced fluorescence intensity. C. Swept-source optical coherence tomography findings of the white arrow in Figure 1A taken with Triton plus ver. 10.18. (Topcon, Tokyo, Japan). The ellipsoid zone was disrupted, and multiple hyper-reflective lesions (arrowheads) beneath the retina were observed. D. UWF fluorescein angiography (FA) and indocyanine-green angiography (ICGA). FA demonstrated early-to-late hyperfluorescence consistent with staining at the sites of exudative lesions, while ICGA showed corresponding hypofluorescent spots due to blocked fluorescence.

A 46-year-old woman noticed blurred vision in her right eye two weeks after receiving the fourth dose of a COVID-19 vaccine (BNT162b2 mRNA vaccine, Pfizer-BioNTech, New York, NY, USA) and visited a local eye clinic. As decreased VA in the right eye was detected, she was referred to our hospital for further examination and treatment. The patient had no history of either eye disease or systemic diseases. Approximately two weeks after receiving her fourth dose of the COVID-19 vaccine, the patient developed blurred vision in the right eye and consulted a local ophthalmologist, where reduced VA was noted. The patient was subsequently referred to our hospital. At the initial visit, the best-corrected visual acuity (BCVA) was 0.7 in decimal units in the right eye and 1.5 in the left eye, respectively. Intraocular pressure was 17 mmHg in both eyes. Anterior segment examination revealed anterior chamber cells in the right eye. Vitreous examination showed vitreous cells and vitreous opacity, and fundus examination revealed multiple white exudative lesions in the right eye (Figure 1A).

**Figure 2:**
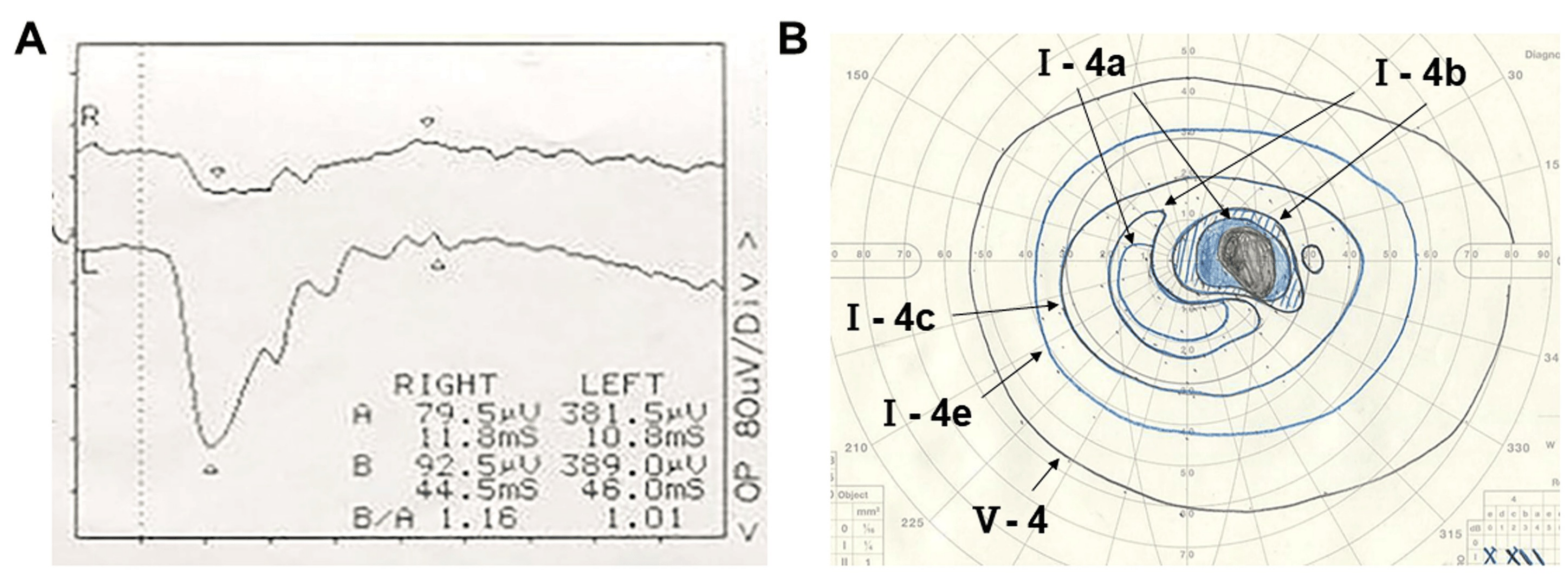
Baseline functional findings A. Full-field electroretinography revealed reduced amplitudes of both a- and b-waves in the right eye. B. Goldman perimetry visual field test; a central scotoma was demonstrated in the right eye.

Fundus autofluorescence (FAF) exhibited a slightly reduced fluorescence intensity corresponding to the white exudative spots (Figure 1B). Optical coherence tomography (OCT) showed disruption of the ellipsoid zone (EZ) and multiple hyper-reflective lesions beneath the retina (Figure 1C). Fluorescein angiography (FA) demonstrated early-to-late hyperfluorescence consistent with staining at the sites of exudative lesions (Figure 1D), while indocyanine green angiography (ICGA) showed corresponding hypofluorescent spots suggestive of blocked fluorescence (Figure 1D). Full-field electroretinography (ERG) revealed reduced amplitudes of both a- and b-waves in the right eye (Figure 2A).

Goldman perimetry demonstrated a central scotoma in the right eye (Figure 2B).

Serologic tests for cytomegalovirus, herpes simplex virus, varicella-zoster virus, hepatitis B antigen, hepatitis C antibody, *Treponema pallidum* latex agglutination, rapid plasma reagin, human immunodeficiency virus, human herpesvirus 6, 7, and 8, *Mycobacterium tuberculosis*, *Toxoplasma gondii*, adenovirus, bacterial 16S rRNA, fungal 28S rRNA, *Toxocara*, *Chlamydia*, *Bartonella*, *Acanthamoeba*, *Candida albicans*, *Candida glabrata*, *Candida krusei*, *Aspergillus*, *Cryptococcus*, and *Fusarium *were negative.

Treatment

**Figure 3:**
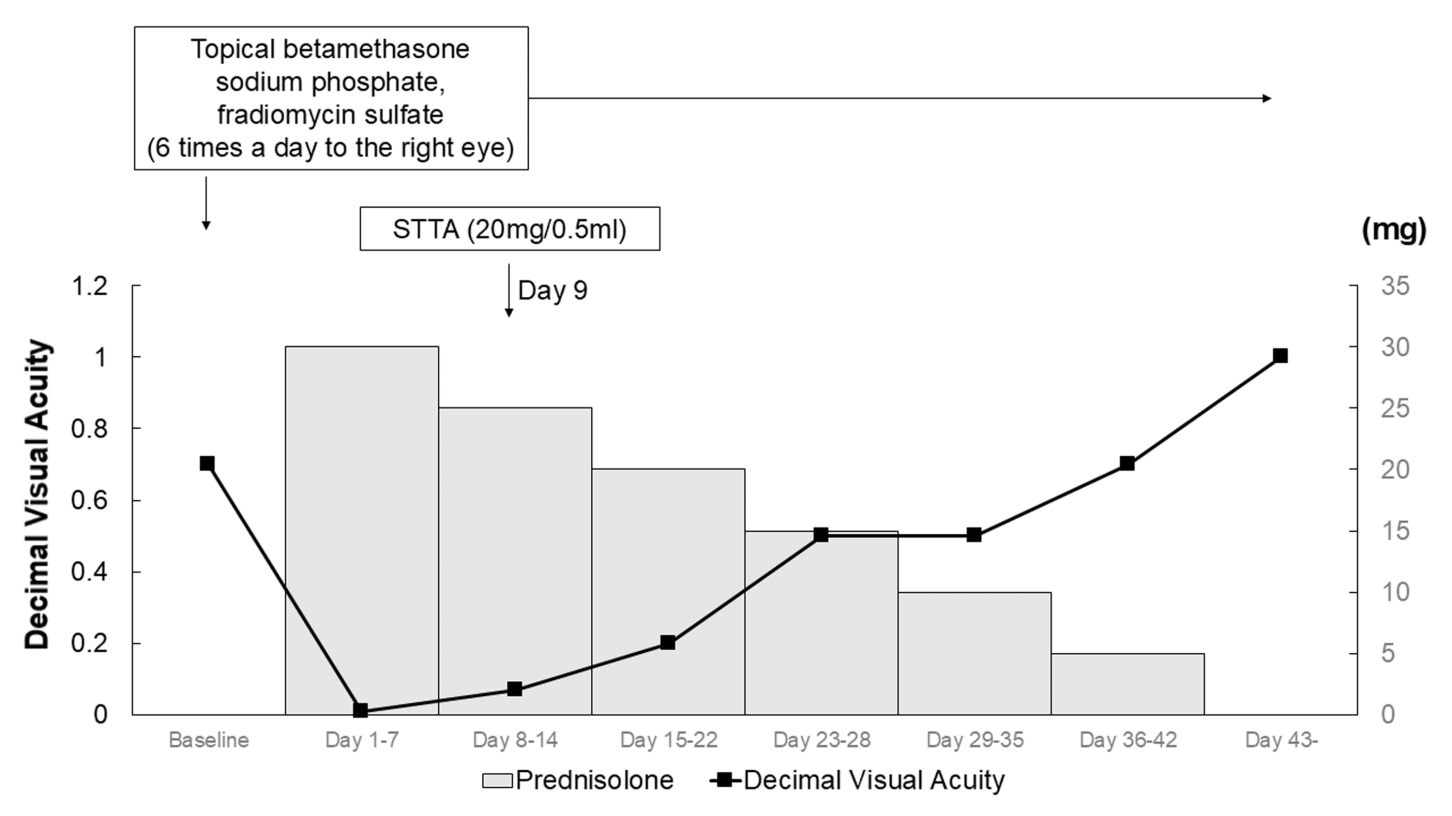
Treatment details and visual acuity progression Baseline best-corrected visual acuity (BCVA) in decimal units was 0.7. Topical betamethasone sodium phosphate and fradiomycin sulfate were administered six times a day to the right eye. The day after the initial visit, the BCVA in the right eye had rapidly decreased to 0.01. She was hospitalized, and oral prednisolone 30 mg/day was initiated. The prednisolone dose was tapered by 5 mg each week. On day 9 of treatment, a sub-Tenon’s injection of triamcinolone acetonide (STTA) was administered. After the treatment, the BCVA gradually improved and recovered to 1.0 on Day 43.

Topical betamethasone, sodium phosphate, and fradiomycin sulfate were administered six times a day to the right eye; however, the day after the initial visit, the BCVA in the right eye had rapidly decreased to 0.01 (Figure 3).

Then, the patient was hospitalized, and oral prednisolone 30 mg/day was initiated (Figure 3). The prednisolone was tapered by 5 mg each week. On day 9 after the treatment, a sub-Tenon’s capsule injection of triamcinolone acetonide (20mg/0.5ml) was administered.

**Figure 4:**
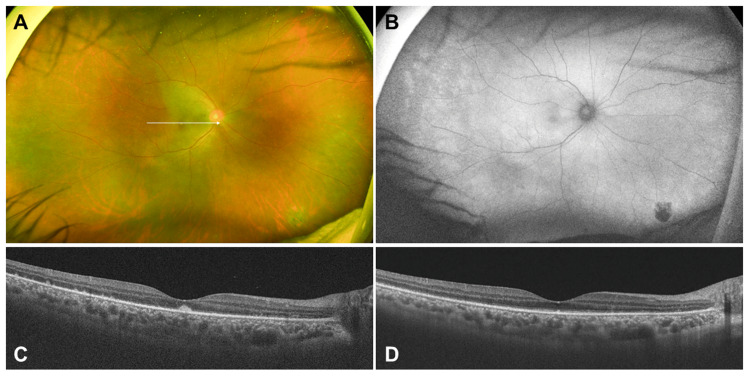
Ocular findings after the treatment A. Ultra-widefield fundus (UWF) photograph on the 10^th^ day after treatment. Vitreous cells and vitreous opacity almost disappeared. Multiple white exudative lesions in the right eye were also decreased. B. UWF fundus autofluorescence on the 10^th^ day after treatment. The slightly hypofluorescent spots had also nearly disappeared. C. Swept-source optical coherence tomography (SSOCT) findings of the white arrow in Figure 4A. Multiple hyper-reflective lesions beneath the retina were decreased. D. SSOCT findings one month after treatment of the white arrow in Figure 4A. Multiple hyper-reflective lesions beneath the retina almost disappeared, and the ellipsoid zone was partially recovered.

By day 10, vitreous opacity had improved, and the white lesions had decreased (Figures 4A, 4B), although EZ disruption persisted on the OCT image (Figure 4C).

By day 18, the BCVA had improved to 0.2, and the white lesions continued to resolve (Figure 4B). OCT showed a decrease in subretinal hyper-reflective lesions, although residual EZ damage remained (Figure 4C). One month later, partial restoration of the EZ was observed (Figure 4D).

**Figure 5:**
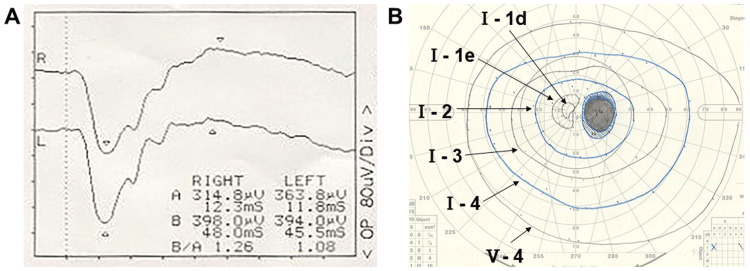
Functional findings two months after treatment A. Full-field electroretinography showed that the a- and b-waves in the right eye had almost normalized. B. Goldman perimetry visual field test; the central scotoma had disappeared. The Marriott’s blind spot was still slightly enlarged.

ERG findings had returned to near-normal (Figure 5A).

**Figure 6:**
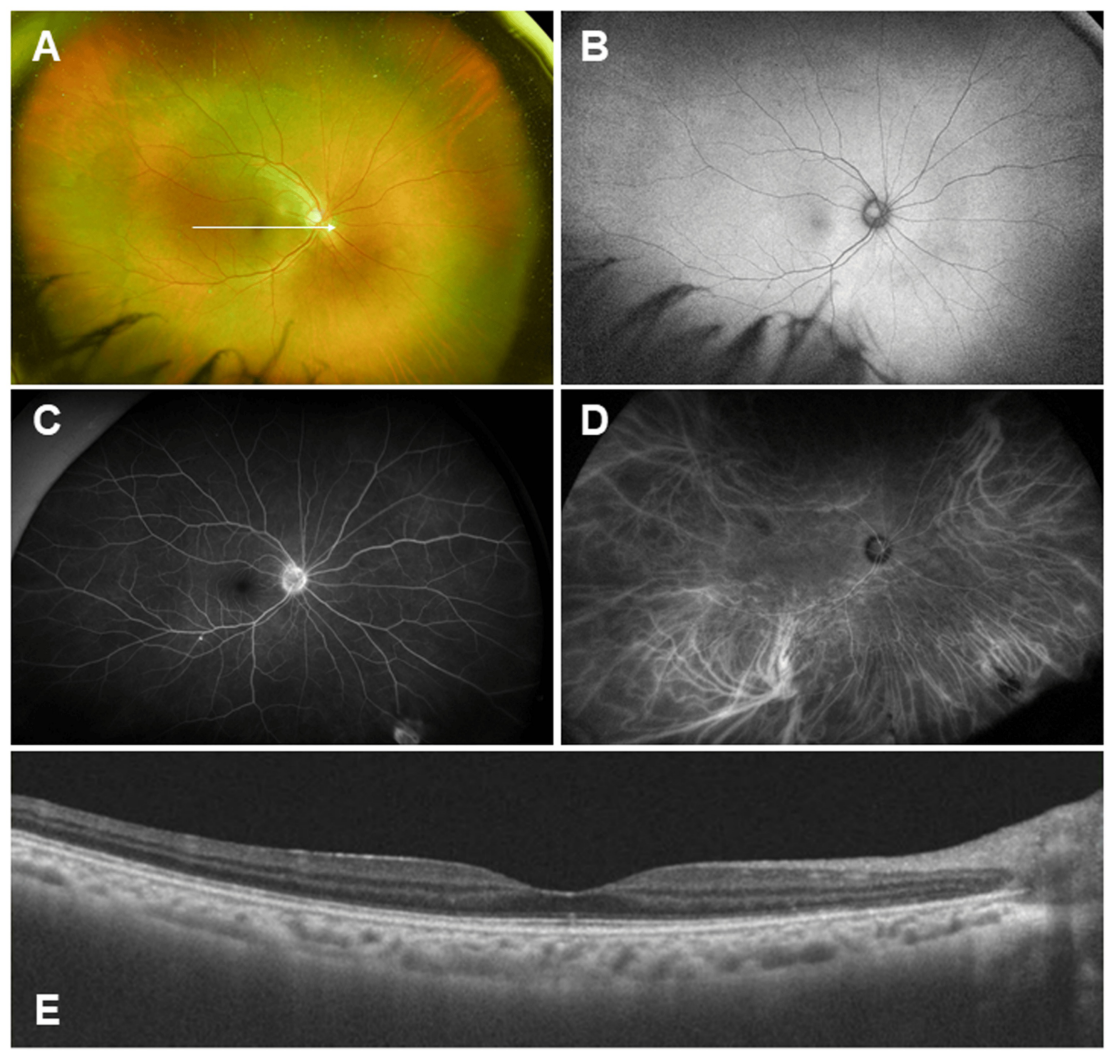
Ocular findings four months after the treatment A. Ultra-widefield (UWF) fundus photograph; B. UWF fundus autofluorescence; C. UWF fluorescein angiography (FA); D. UWF indocyanine-green angiography (ICGA); E. Swept-source optical coherence tomography of the white arrow in Figure 6A. All findings from the fundus, optical coherence tomography, and FA/ICGA had almost normalized.

Two months later, Goldman perimetry revealed persistent enlargement of the Mariott’s blind spot but resolution of the central scotoma (Figure 5B). Subsequently, the white exudative lesions had completely resolved without scarring (Figures 6A, 6B).

The hyperfluorescence seen on FA had resolved (Figure 6C), and ICGA showed no remaining hypofluorescent spots (Figure 6D). OCT demonstrated full recovery of the EZ, and the subretinal hyper-reflective lesions had almost disappeared (Figure 6E). The BCVA improved to 1.0. Because the white fundus lesions resolved without scarring and visual function recovered, the patient was diagnosed with MEWDS.

## Discussion

Vaccination with mRNA COVID-19 vaccines against COVID-19 began worldwide in late 2020. Since then, an increasing number of adverse events following vaccination have been reported, and accumulating evidence has described vaccine-associated uveitis [3-5]. Reported ophthalmic adverse effects include anterior uveitis and uveitic diseases such as Vogt-Koyanagi-Harada disease [5,15].

We report a case of MEWDS that was considered to be triggered by COVID-19 vaccination. Although the patient presented severe visual impairment, the visual prognosis was favorable following appropriate treatment. MEWDS is classified among the white dot syndromes, a group of disorders characterized by multiple scattered white lesions in the posterior fundus. The differential diagnosis includes multifocal choroiditis (MFC), punctate inner choroidopathy (PIC), acute posterior multifocal placoid pigment epitheliopathy (APMPPE), acute retinal pigment epithelitis (ARPE), birdshot chorioretinopathy (BCR), and acute macular neuroretinopathy (AMN). Among these, APMPPE typically presents with bilateral involvement and multiple large yellowish-white placoid lesions. A characteristic finding of APMPPE is the “fluorescence reversal” on FA, showing early hypofluorescence followed by late hyperfluorescence [16,17]. These features were not consistent with the current case.

ARPE does not usually present with anterior segment or vitreous inflammation. Its posterior fundus shows white lesions surrounded by a yellowish-white halo. FA demonstrates central hypofluorescence with peripheral hyperfluorescence [18,19]. PIC commonly occurs in myopic eyes and is often bilateral, without inflammatory cells in the anterior chamber or vitreous. Small punctate subretinal white lesions appear scattered in the posterior pole and eventually evolve into pigmented chorioretinal scars, although the overall visual prognosis is generally favorable. Choroidal neovascularization may occasionally develop [20]. The absence of inflammatory cells in PIC and ARPE distinguishes these conditions from the present case.

MFC is characterized by yellowish-white lesions at the level of the RPE extending from the posterior pole to the midperiphery. These lesions evolve into pigmented scars over time, and the disease often becomes chronic with recurrent episodes, frequently resulting in a poor visual prognosis [21,22].

BCR rarely presents with anterior segment inflammation, although mild anterior uveitis may occasionally be observed. On fundus examination, characteristic cream-colored, oval choroidal lesions are typically evident; however, these findings may be subtle or absent in the early stages of the disease. As BCR progresses, the lesions tend to coalesce and form a linear pattern along the retinal veins. Eventually, the exudative lesions evolve into white atrophic areas without pigment deposition, producing the characteristic "birdshot" appearance [23,24]. FA findings are variable: hypofluorescence may be observed in the early phase, while subtle late staining can be seen in some cases [23]. However, these angiographic features were not consistent with those observed in the present case.

AMN presents with acute visual loss or paracentral scotomas in one or both eyes [25]. In this respect, particularly the presence of a preceding inflammatory process, AMN is consistent with the clinical course observed in the present case. In addition, cases of AMN occurring concurrently with MEWDS have been reported [26], and thus AMN should be considered in the differential diagnosis. However, AMN is characterized funduscopically by cloverleaf-shaped or wedge-shaped reddish-brown lesions surrounding the fovea [25], and therefore, its fundus findings are not consistent with those observed in the present case.

In our case, despite the presence of rapid visual deterioration and reduced ERG responses, oral corticosteroid therapy combined with a sub-Tenon’s injection led to a complete disappearance of the lesions without scarring and recovery of visual function. Considering the resolution of symptoms and clinical findings, unilateral involvement, and the patient’s sex, MEWDS was determined to be the most appropriate final diagnosis.

MEWDS was first described by Jampol et al. in 1984 as an acute, idiopathic, and typically unilateral cause of visual disturbance [13]. It presents with small gray-white dots at the outer retina and RPE, along with optic disc edema and vitreous cells. In the acute phase, FA shows early hyperfluorescence corresponding to the lesions, which persists into the late phase without expansion. ICGA is characterized by hypofluorescent spots in the late phase [27,28]. The prognosis of MEWDS is generally excellent; lesions typically resolve spontaneously within three months, and patients usually regain normal visual acuity and visual fields under observation alone [14,29]. Recurrence is relatively rare, occurring in approximately 14% of cases [30]. Several reports have described treatment with corticosteroids, which may accelerate visual recovery within several weeks [31,32].

Approximately 30% of patients with MEWDS report viral prodromal symptoms [33]. MEWDS has been reported following rabies, human papillomavirus, hepatitis A and B, meningococcal, yellow fever, and influenza vaccinations [34,35]. The precise pathogenesis remains unclear; however, an immune-mediated mechanism is suspected, in which viral infection may trigger disease in genetically predisposed individuals. The growing number of MEWDS cases reported after various vaccinations further supports this theory [6,36]. Recent hypotheses suggest that immune-mediated mechanisms occur at the outer retina [37], choriocapillaris, or inner choroid [38] in susceptible individuals. Proposed mechanisms include molecular mimicry, direct antigen-mediated cellular or humoral immune responses, and adjuvant-mediated inflammation, ultimately leading to deposition of vaccine-related antigens in the retina and triggering MEWDS [7,34]. Further investigation is needed.

Several cases of MEWDS following COVID-19 vaccination have been reported. Yasuda et al. described a 67-year-old woman who developed MEWDS the day after her second COVID-19 vaccination [8]. Bouhout et al. reported three cases of women aged 28 and 26 years and a 27-year-old man who developed MEWDS after COVID-19 vaccination [9]. Ng et al. reported a 28-year-old woman who developed MEWDS following COVID-19 vaccination and subsequently developed contralateral MEWDS one year later during COVID-19 infection [10]. All of these cases recovered spontaneously without therapeutic intervention.

Regarding steroid-treated cases, Tomishige et al. described the case of a 38-year-old woman who developed MEWDS two weeks after her first COVID-19 vaccination and was treated with oral prednisolone 80 mg followed by tapering, with visual recovery achieved after four weeks [7]. Ting et al. reported the case of a 69-year-old woman who developed MEWDS after COVID-19 vaccination and subsequent infection; she was treated with oral prednisolone 40 mg for one week followed by weekly 10 mg tapering, with visual recovery at three months [6]. Soifer et al. described the case of a 31-year-old woman who developed MEWDS two weeks after her second vaccination and recovered within three months without intervention; however, she experienced recurrence after receiving a booster dose 12 months later, which improved following a tapering course of oral prednisolone 60 mg over eight weeks [11]. Inagawa et al. reported the case of a 30-year-old woman treated with betamethasone sodium phosphate/fradiomycin sulfate 0.1% three times daily for two months after developing MEWDS 13 days post vaccination, with improvement noted at two months [12]. In all COVID-19 vaccine-related MEWDS cases reviewed, visual outcomes were ultimately favorable, either spontaneously or with steroid therapy.

The mechanisms by which SARS-CoV-2 contributes to the development of retinal diseases such as MEWDS remain under investigation. One proposed mechanism is impaired capillary perfusion [36]. In COVID-19 patients, vascular endothelial damage caused by both the virus and inflammatory cytokines results in severe capillary perfusion abnormalities. SARS-CoV-2 targets endothelial cells via angiotensin-converting enzyme 2 receptors, causing significant morphological changes. Endothelial dysfunction leads to exudation and accumulation of inflammatory cells beneath the RPE. Accumulated cells exert stretching forces on the RPE, contributing to its thinning and disruption. Microcirculatory impairment results in capillary congestion, microthrombi formation, and pericyte damage, all essential for maintaining capillary integrity and barrier function. The breakdown of the peripapillary microcirculation is thought to contribute to the development of MEWDS [6,36].

## Conclusions

In this report, we describe a case of MEWDS that developed after COVID-19 vaccination. Because the patient exhibited marked visual impairment, we initiated systemic corticosteroid therapy combined with a sub-Tenon’s injection, which resulted in a favorable visual outcome. Although the patient did not experience any viral prodromal symptoms, the temporal association with COVID-19 vaccination raises the possibility that vaccine-induced inflammation may have triggered the onset of MEWDS.

The relationship between COVID-19 and MEWDS remains under investigation; however, if the hypothesis that inflammatory disruption of capillary barrier function contributes to disease pathogenesis is correct, the numerous case reports, including the present case, in which corticosteroid treatment led to visual improvement, may support this proposed mechanism.
